# Supplementary material for: Genome-wide mapping and cryo-EM structural analyses of the overlapping tri-nucleosome composed of hexasome-hexasome-octasome moieties
Source: Commun Biol. 2024 Jan 8;7:61. doi: 10.1038/s42003-023-05694-1 (PMC10774305; doi:10.1038/s42003-023-05694-1)
Supplement: Supplementary file 2 — Supplementary Information [file 42003_2023_5694_MOESM2_ESM.docx]

Supplementary Information

**Genome-wide mapping and cryo-EM structural analyses of the overlapping tri-nucleosome composed of hexasome-hexasome-octasome moieties**

Masahiro Nishimura^#1†^, Takeru Fujii^#2^, Hiroki Tanaka^1††^, Kazumitsu Maehara^2^, Ken Morishima^3^, Masahiro Shimizu^3^, Yuki Kobayashi^1^, Kayo Nozawa^1†††^, Yoshimasa Takizawa^1^, Masaaki Sugiyama^3^, Yasuyuki Ohkawa^2^*, and Hitoshi Kurumizaka^1^*

^1^ Laboratory of Chromatin Structure and Function, Institute for Quantitative Biosciences, The University of Tokyo, 1-1-1 Yayoi, Bunkyo-ku, Tokyo 113-0032, Japan.

^2^ Division of Transcriptomics, Medical Institute of Bioregulation, Kyushu University, 3-1-1 Maidashi, Higashi, Fukuoka 812-0054, Japan.

^3^ Institute for Integrated Radiation and Nuclear Science, Kyoto University, Kumatori, Sennan-gun, Osaka 590-0494, Japan.

^#^ Contributed equally.

†Present address: Epigenetics and Stem Cell Biology Laboratory, National Institute of Environmental Health Sciences, Research Triangle Park, 111 TW Alexander Drive, NC, 27707, USA.

††Present address: Department of Structural Virology, National Center for Global Health and Medicine, 1-21-1 Toyama, Shinjuku-ku, Tokyo 162-8655, Japan.

†††Present address: School of Life Science and Technology, Tokyo Institute of Technology, 4259 Nagatsuta-cho, Midori-ku, Yokohama, Kanagawa 226-8501, Japan.

Correspondence should be addressed to Y.O. (yohkawa@bioreg.kyushu-u.ac.jp) and H.K. (kurumizaka@iqb.u-tokyo.ac.jp)

Running title: Cryo-EM structure of the overlapping tri-nucleosome

**Supplementary Figures**

**
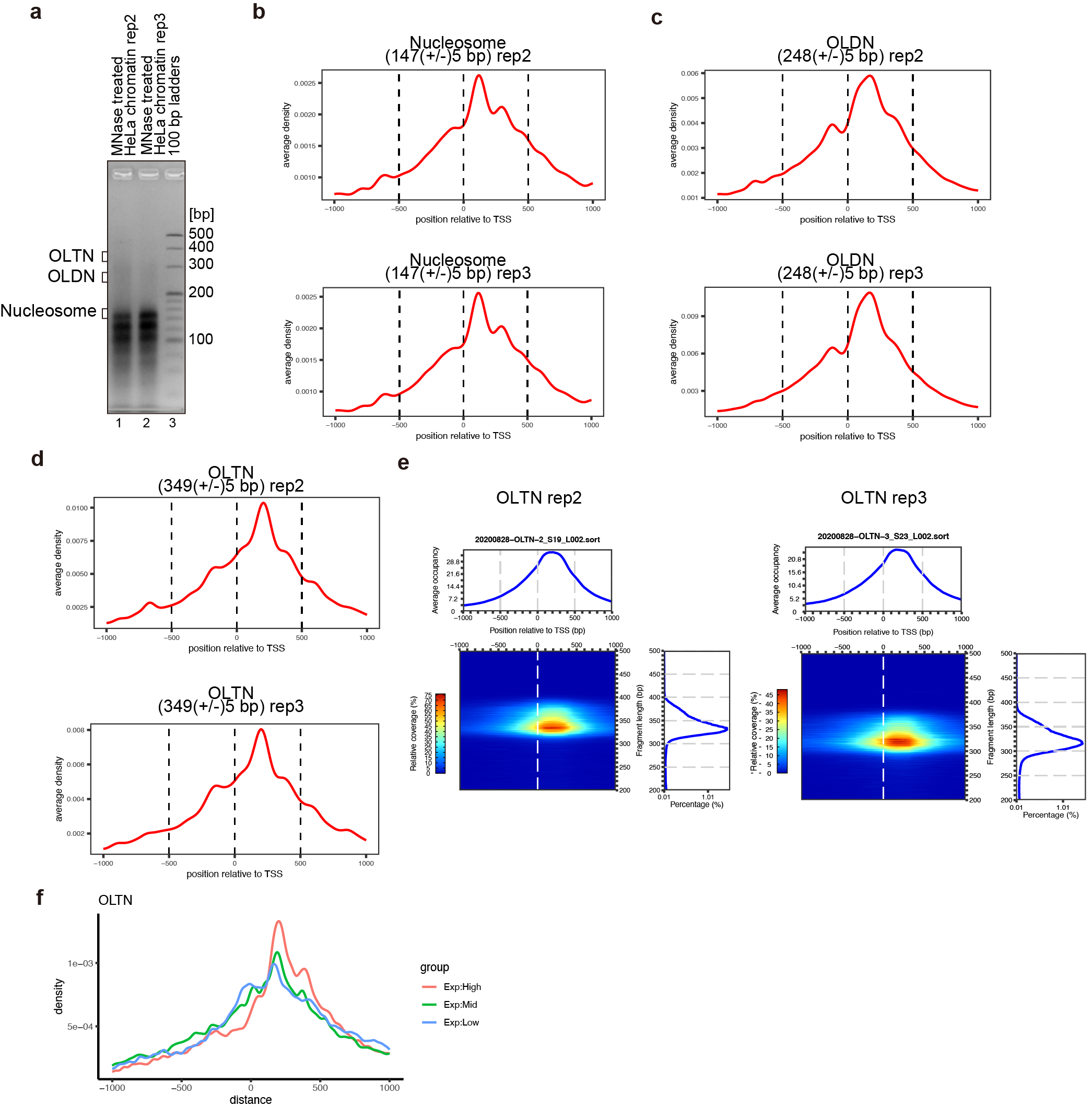
**

**Supplementary Fig. 1: Reproducibility of the MNase-seq analyses, related to Fig. 1.**

**a,** Replicated MNase-seq experiments using extensively digested HeLa chromatin, related to Fig. 1a. The genomic DNA fragments protected from MNase digestion by nucleosome, overlapping di-nucleosome (OLDN), and overlapping tri-nucleosome (OLTN) formation were analyzed by agarose gel electrophoresis with ethidium bromide staining. The DNA fragments with lengths of about 140~150 bp, 200-300 bp, and 300-400 bp, which may correspond to nucleosomes, OLDNs, and OLTNs, respectively, were extracted from the agarose gel and subjected to next generation sequencing analysis. **b-d,** Aggregation plots of the DNA fragments corresponding to nucleosomes for the replication experiments (**b**), OLDNs (**c**), and OLTNs (**d**) relative to TSSs. The reads with the indicated lengths extracted from the sequencing data were mapped between a +/- 1 kb range around TSSs. **e,** Heatmap representation of the aggregation plot analysis of the overlapping tri-nucleosome expanded by the DNA fragment length for the replication experiments. The histogram of the DNA fragment length is presented in the right panel. **f,** Aggregation plots of the DNA fragments corresponding to OLTN (average of three replicates of MNase-seq). The nucleosome densities at genes' TSS of High/Mid/Low expression were shown. The genes were divided into three equal sized group according to the expression levels in HeLa cells (average TPM of two replicates; ENCFF516HMD, ENCFF543ZTH). The genes with no expression were excluded.


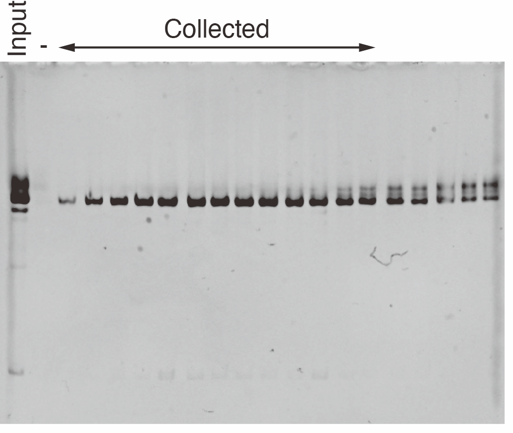


**Supplementary Fig. 2: Fractionation of the OLTN after Prep Cell**

The OLTN after Prep Cell was analyzed by non-denaturing polyacrylamide gel electrophoresis with ethidium bromide staining.


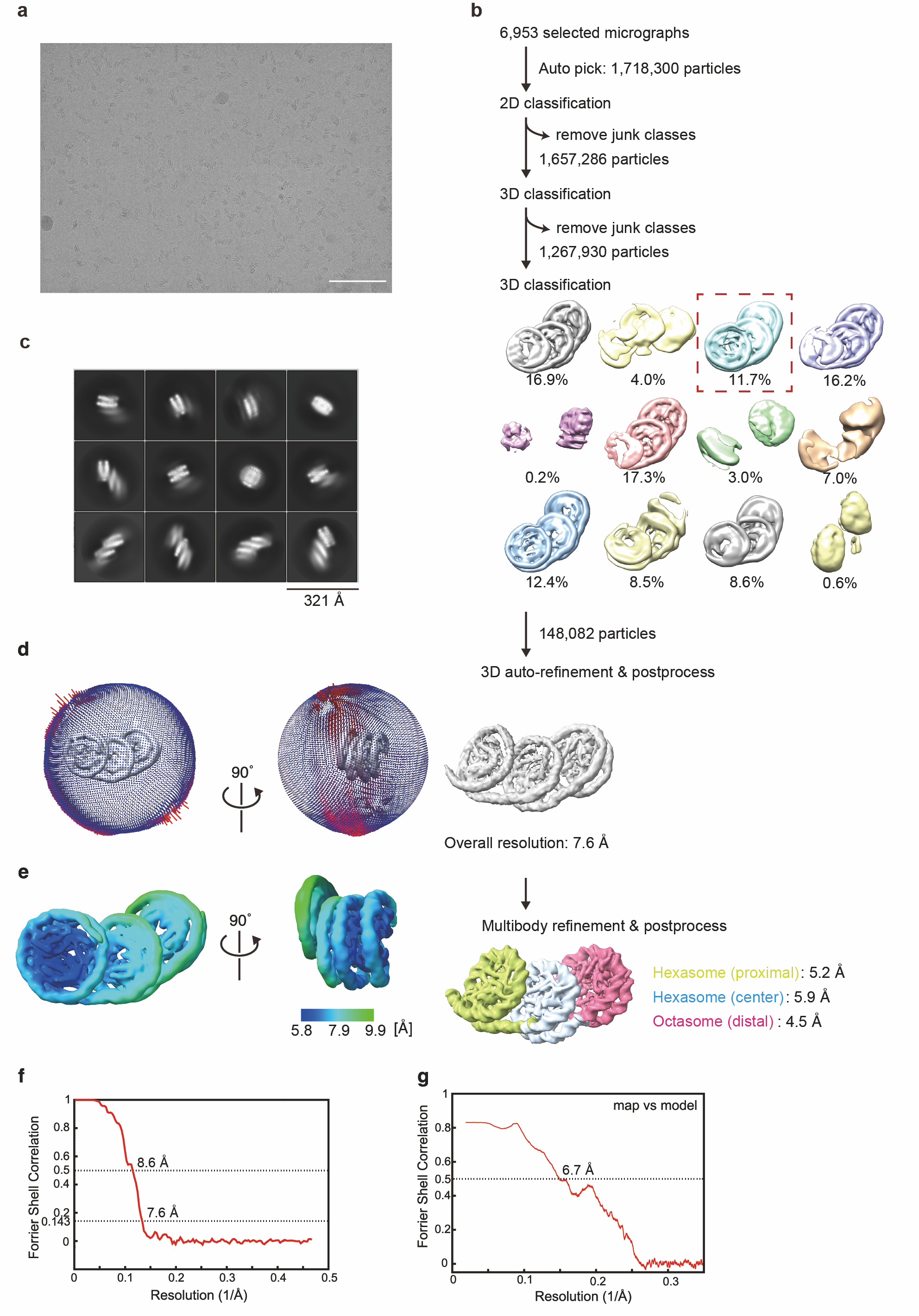


**Supplementary Fig. 3: Cryo-EM analysis of the OLTN**

**a,** Representative micrograph from 6,953 images in the dataset. Scale bar indicates 100 nm. **b,** Flowchart of the image processing. **c,** Representative 2-dimensional class averages with a 321 Å^2^ box size. **d,** Euler angular distribution plots of the overall reconstruction. **e,** Local resolution map of the overall reconstruction of the overlapping tri-nucleosome. The local resolution is shown by the colors indicated in the bar on the right. **f,** The Fourier shell correlation four the 3D reconstruction. The resolutions of Fourier shell correlation criteria 0.143 and 0.5 are shown. **g,** The Fourier shell correlation curve of map to model.

**
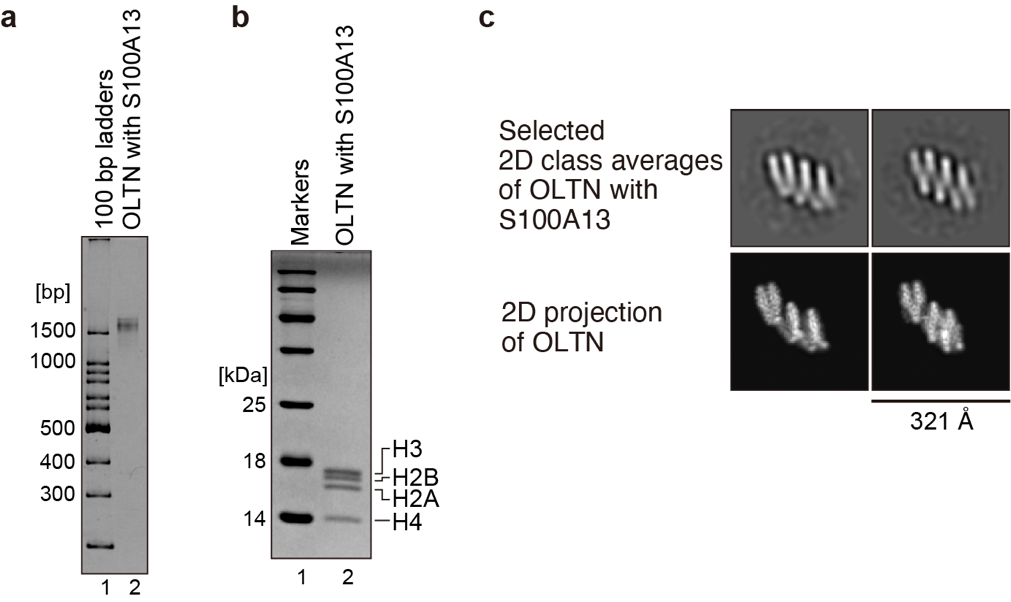
**

**Supplementary Fig. 4: Reconstitution of the OLTN with a native DNA sequence**

**a.** The OLTN containing native DNA sequence (OLTN with S100A13 DNA) was analyzed by non-denaturing polyacrylamide gel electrophoresis with ethidium bromide staining. **b,** The histone content of the OLTN was analyzed by SDS-PAGE with Coomassie Brilliant Blue staining. c, Representative 2-dimensional class averages of the OLTN containing native DNA sequence (top) and the 2D projection of the OLTN structure (bottom) were shown. The box sizes are 321 Å^2^.

**
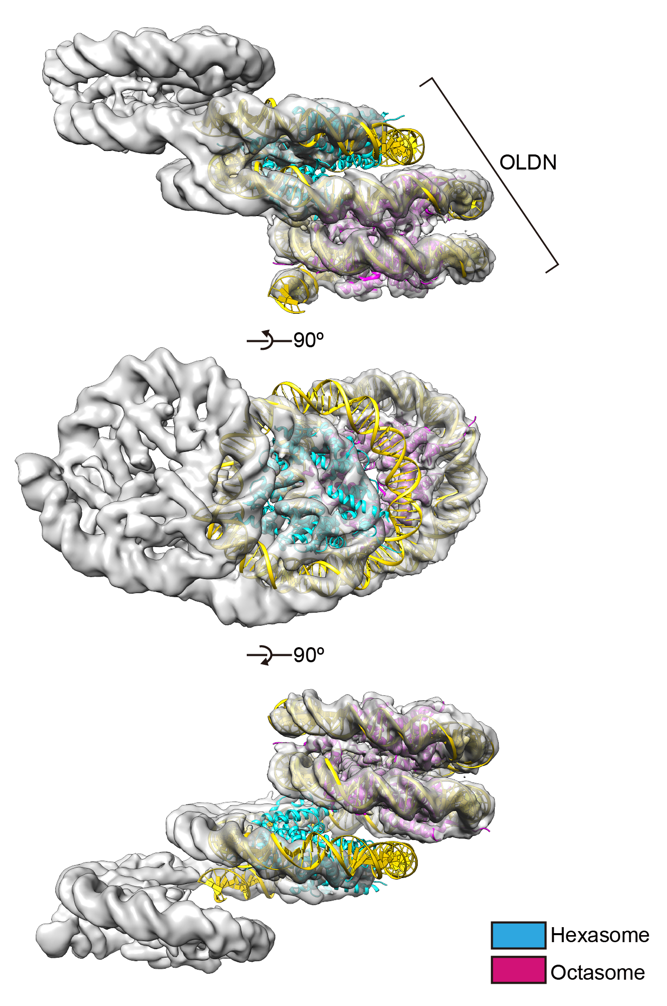
**

**Supplementary Fig. 5: Comparison the cryo-EM structure of the OLTN with the crystal structure of the OLDN**

The crystal structure of the OLDN (PDB: 5GSE) is docked into the cryo-EM map of the OLTN.


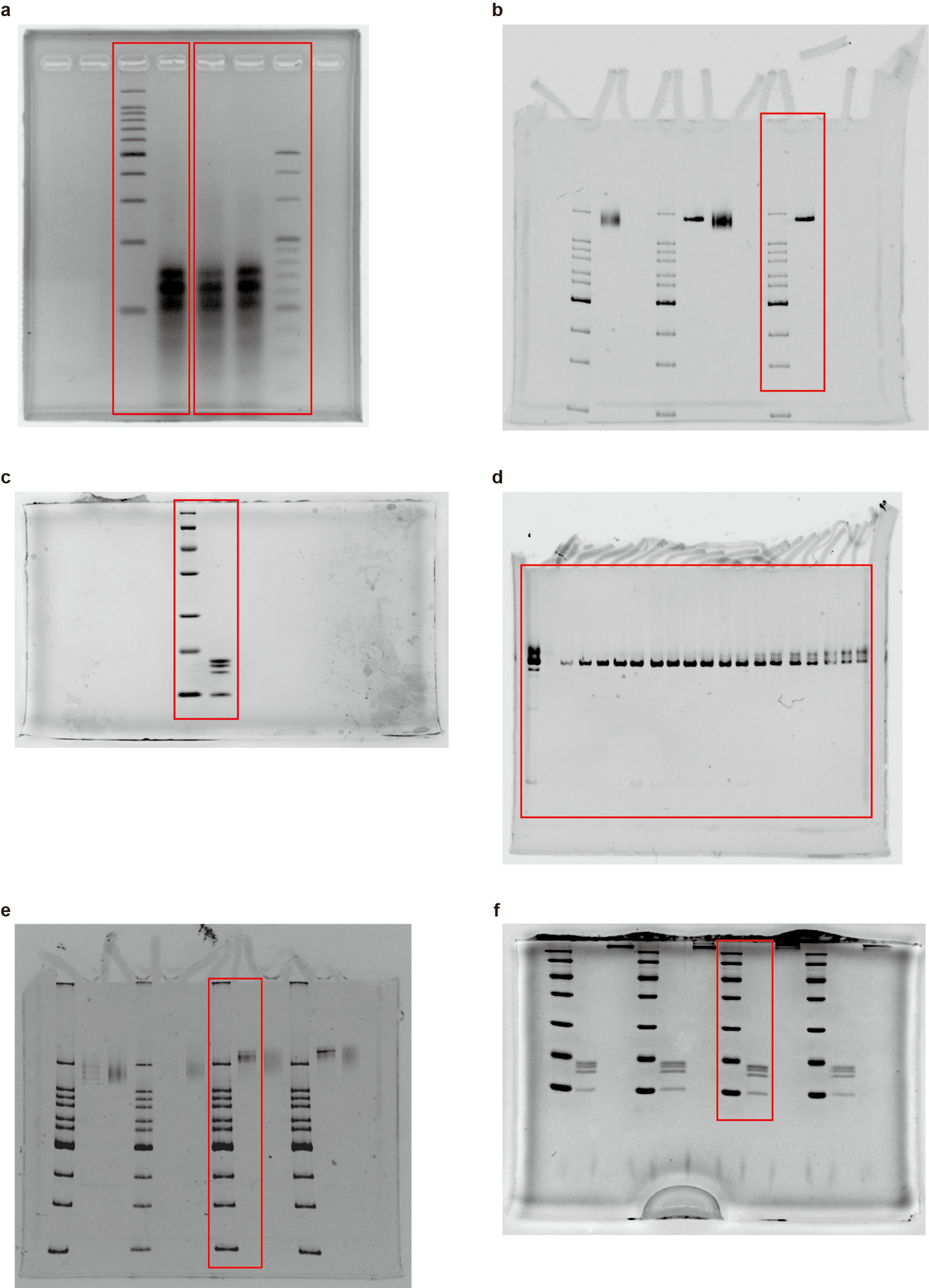


**Supplementary Fig. 6: Uncropped images related to Fig. 1, Fig. 2, Supplementary Fig. 1, Supplementary Fig. 2, and Supplementary Fig. 4.**

**a.** Uncropped image related to Fig. 1a (left rectangle) and Supplementary Fig. 1a (right rectangle). **b.** Uncropped image related to Fig. 2b. **c.** Uncropped image related to Fig. 2c. **d.** Uncropped image related to Supplementary Fig. 2. **e.** Uncropped image related to Supplementary Fig. 4a. **f.** Uncropped image related to Supplementary Fig. 4b. The red rectangles indicate the lanes shown in the figures.
